# Whole exon screening of *SLC2A4* gene and the association of rs5435 with type 2 diabetes in a Bangladeshi case-control study

**DOI:** 10.1016/j.jgeb.2025.100534

**Published:** 2025-07-10

**Authors:** Mohammad Mamunur Rashid, Mohammad Sayem, Maisha Adiba, Abdullah Al Saba, A.H.M. Nurun Nabi, Tahirah Yasmin

**Affiliations:** a Laboratory of Population Genetics, Department of Biochemistry and Molecular Biology, University of Dhaka, Bangladesh

**Keywords:** T2D, GLUT4, SLC2A4, SNPs, Rs5435, *In silico* analyses

## Abstract

Type 2 diabetes (T2D) is a multifaceted disease influenced by both genetic and environmental factors, posing significant global health challenges. Polymorphisms in the *SLC2A4* gene encoding the glucose transporter 4 (GLUT4), have been linked to insulin resistance and T2D. This study, therefore, aims to identify and analyze genetic variants within the *SLC2A4* gene and determine their association with T2D risk through a Bangladeshi case-control study. A total of 239 individuals were enrolled in the study, including 127 T2D patients and 112 controls. All 11 exons of the SLC2A4 gene were sequenced in 20 individuals. Exons 2, 3, 4, and 5 of GLUT4 were then sequenced in an additional 99 participants. For the remaining 120 participants, a TaqMan probe-based RT-PCR was conducted to genotype rs5435. Four genetic variants were initially identified, including one synonymous variant (rs5435) in exon 4. There was no significant difference in the allele frequency of the wild-type allele T and variant allele C between the two groups (OR: 1.31, p = 0.13). Genotypic distribution revealed that individuals who were either heterozygous or homozygous for the variant allele are at an increased risk for developing T2D compared to those who are homozygous for the non-risk allele (p = 0.042), suggesting that the SNP could be considered to have a dominant effect on the likelihood of developing T2D. Additionally, *in silico* analysis revealed that the rs5435 variant introduces an additional loop in the mRNA secondary structure, which is energetically less stable and therefore may interfere with the protein’s biological function and play a role in the pathogenesis of T2D. However, further studies with larger cohorts are necessary to validate the impact of rs5435 on the risk of T2D in our population.

## Highlights

• Rs5435 in GLUT4 is present in the Bangladeshi population.
• The C allele of rs5435 was more prevalent in T2D patients than in controls.
• The dominant model shows a statistically significant association with T2D.
• rs5435 variant introduces an additional loop in the mRNA secondary structure.

## 1 Introduction

Type 2 Diabetes Mellitus (T2D) is a chronic metabolic disorder and a significant global health concern, marked by insulin resistance and impaired insulin secretion that result in elevated blood glucose levels. It is the most prevalent form of diabetes, accounting for approximately 90–95 % of all diabetes cases. The global burden of diabetes is substantial and rising. In 2025, the International Diabetes Federation estimated 590 million diabetic individuals worldwide, a number projected to rise to 853 million by 2050^1^.

In Asian countries, particularly South Asia, diabetes prevalence is notably high, often linked to rapid socioeconomic changes and underlying genetic susceptibility. Bangladesh, as a South Asian nation, reflects this trend with a significant and steadily rising burden of diabetes. In 2021, the prevalence of diabetes in Bangladesh was 14.2 %, with projections indicating an increase to 15 % by 2030^1^. This trend places a considerable strain on the country's healthcare system and highlights the urgent need for in-depth understanding and effective management strategies.

Genetic factors play a critical role in the susceptibility to T2D. Among the genes implicated in T2D, the *SLC2A4* gene, encoding the GLUT4 protein, is particularly important. GLUT4 is a primary insulin-responsive glucose transporter in adipose tissue and muscle, and is integral to glucose homeostasis. GLUT4 holds a key position in the pathophysiology of T2D as impaired expression or defective translocation of GLUT4 to the cell membrane inhibits glucose uptake into cells for energy production. Glucose, due to its large and polar nature, cannot diffuse through cell membranes on its own and instead relies on specific glucose transporters. Members of the GLUT family share structural features, including 12 transmembrane domains and cytoplasmic amino- and carboxy-termini. Genetic variations within these transporters, particularly single nucleotide polymorphisms (SNPs), are pivotal for understanding the genetic basis of various diseases, including T2D. For instance, variants that reduce GLUT4 expression or impair its insulin responsiveness may increase the risk of insulin resistance and, ultimately, T2D. Additionally, SLC2A4 polymorphisms may affect GLUT4′s efficiency in translocating to the cell membrane or its function once localized there. Variants that disrupt these processes can impair glucose uptake, leading to elevated blood sugar levels—a hallmark of T2D pathogenesis.

SNPs are typically classified based on their impact on proteins: non-synonymous SNPs alter amino acids, while synonymous ones do not. Numerous studies across diverse regions and ethnic populations have identified correlations between *SLC2A4* gene polymorphisms and diseases including diabetes mellitus. These studied populations include Euro-Brazilians^2^, Mexican Americans^3^ and Caucasian ^4^, British and Welsh whites^5^, Japanese^6^, African Americans^7^, South Indians^8^, ^9^, Polish^10^, and Chinese^11^.

In this work whole exon sequencing of the *SLC2A4* gene was performed to find out any novel variants in the Bangladeshi population. Much of the research on *SLC2A4* gene polymorphisms and T2D risk has focused on populations of European, East Asian, or African descent. These populations have been well-studied, but genetic research on South Asian populations, including Bangladeshi individuals, is relatively underrepresented although South Asians have been shown to have a higher susceptibility to T2D. Genetic markers linked to specific gene-disease associations exhibit varying frequencies across different populations. Therefore, genetic profiling of functional genes not only aids in the investigation of specific diseases but also enhances our understanding of disease susceptibility within particular populations which, in turn, can lead to personalized medicine. Moreover, it has been suggested that ethnic differences in disease susceptibility arise not solely from genetic variations, but from a complex interplay of genetic factors, environmental influences, diet, and physical activity. Therefore, building a comprehensive database of polymorphisms associated with T2D in the Bangladeshi population is crucial for tailoring medical approaches to the unique needs of our population.

In addition, this study focuses on a specific synonymous SNP, rs5435, within the *SLC2A4* gene, investigating its prevalence and impact on T2D in the Bangladeshi population. Initial exon screening in our study identified several variants, among which rs5435 was the only one with a known association with T2D and other diseases^12^, ^13^. Despite being a synonymous variant, rs5435 has been functionally implicated in the literature, highlighting its potential biological relevance^12^, ^13^ Therefore, this SNP was selected as the primary focus of our study. Previous studies investigating *SLC2A4* have focused on nonsynonymous variants or regulatory variants as they are thought to have a more direct impact on disease risk by causing protein changes or altered gene expression. However, here a synonymous variant in SLC2A4 was focused, suggesting that synonymous variants may have functional relevance by influencing mRNA structure, stability, translation efficiency, or other regulatory aspects. Given the high prevalence of T2D in Bangladesh and the potential role of genetic factors in its pathogenesis, this research provides critical insights into the genetic epidemiology of T2D, which could have implications for the development of targeted interventions and treatments.

## 2 Materials and methods

### 2.1 Study design, sample number calculation and Collection of blood samples

**Fig. 1:**
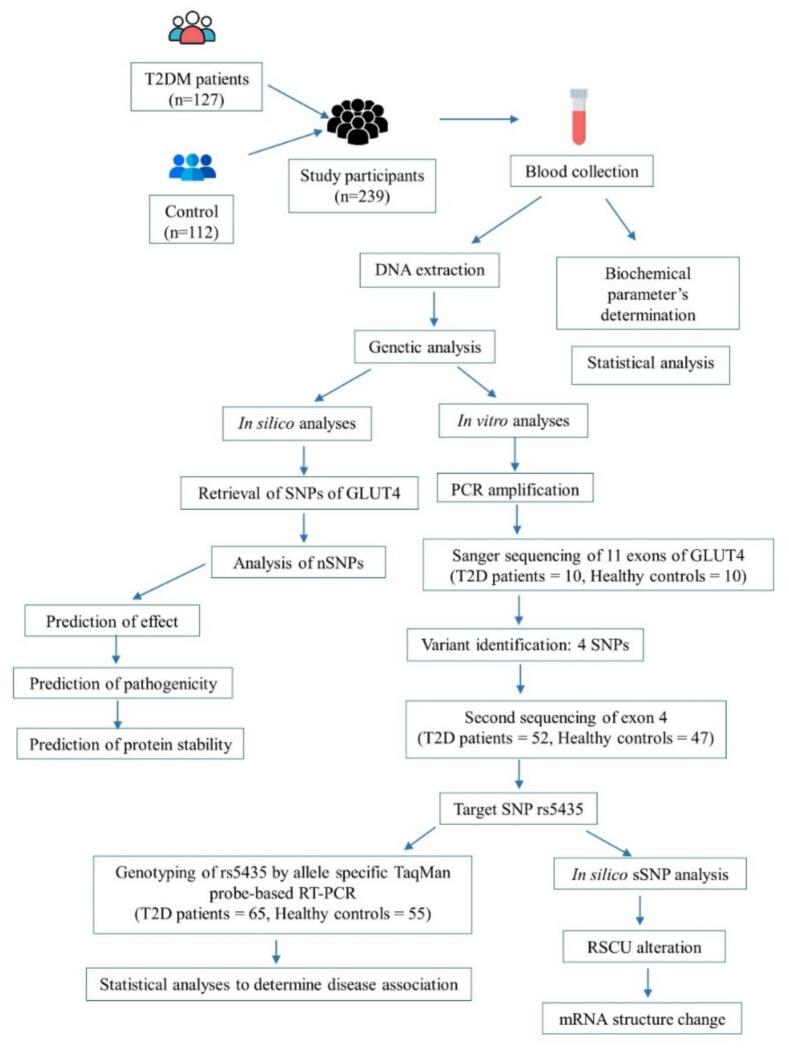
Flowchart illustrating the research activities carried out in this study.

This study was approved by the Ethical Review Committee of the Faculty of Biological Sciences, University of Dhaka, Bangladesh. Fig. 1 illustrates the research strategies and activities undertaken in this study.

The number of samples required to perform the study was calculated using the following formula: n > ln [l − (1 − a)^1/r^] / 2ln (1 − p)^14^, where n = required number of samples, number of alleles (r) is 3 and minimum allele frequency (p) is 0.1 in East Asia according to ALFRED (allele frequency database^15^. Following the above formula, the minimum number of samples required for this study was determined to be 20.

To find out the association of the SNPs of the *SLC2A4* gene with the risk of type 2 diabetes, blood samples were collected from a total of 239 Bangladeshi unrelated individuals. Among the study participants, 127 individuals (male 60, female 67) were diagnosed as type 2 diabetic patients. The remaining participants were healthy controls (male 62, female 50). Type 2 diabetic patients were confirmed using the levels of fasting plasma glucose (>7.0 mmol/L) set by the World Health Organization and recruited from the Bangladesh Institute of Research and Rehabilitation in Diabetes, Endocrine and Metabolic Disorders (BIRDEM) Hospital at Shahbag, Dhaka, Bangladesh. This is a high-volume tertiary center where patients from all regions of Bangladesh are admitted and therefore, the participants’ pool reflects a diverse cross-section of the national population. Levels of plasma glucose were measured using the standard method. Age, gender, height, weight, BMI, systolic and diastolic blood pressure and duration of disease were also recorded in a well-defined questionnaire.

### 2.2 Genetic analysis

#### 2.2.1 DNA extraction

Genomic DNA was extracted from the white blood cells by organic extraction procedure, quantified and finally, the quality was checked according to a previous method^16^.

#### 2.2.2 PCR Amplification, quality control and Sanger sequencing

The targeted region of DNA was PCR amplified. Four sets of primers were designed using a primer designing tool- primer blast (https://www.ncbi.nlm.nih.gov/tools/primer-blast/) to ensure optimum binding with the templates that are presented in Table 1.

**Table 1:** Primers predicted by the NCBI primer designing tool to amplify the whole exon of the *SLC2A4* gene.

Exon no	Position	Size bp	Primer pair		Product length
1	4984–5233	250	P1	FP: GTCCAGACCCGCAGAGTTTA RP: CTGGCTCGCAGAATCCACTT	818 bp
2	6511–6627	117	P2	FP: CCAGTTTCCCTCACCCAACA RP: CACCTGTGGGAGAAGGTTGG	1106 bp
3	6739–6911	173			
4	7004–7128	125			
5	7240–7355	116			
6	7483–7645	163	P3	FP: CTGGGCAGTGGTTAGAGTGG RP: CTGTGTGATGCCCACCTCTG	1849 bp
7	7751–7938	188			
8	8101–8205	105			
9	8354–8455	102			
10	8971–9174	204			
11	9692–11523	1832	P4	FP: CACCTCACTCCGTCAACACC RP: GGTTGAAGGAAGGGAGCCAT	2091 bp

FP = forward primer, RP = reverse primer, bp = base pair.

The PCR conditions were as follows: an initial denaturation at 95 °C for 5 min, followed by 35 cycles of denaturation at 95 °C for 40 s, annealing at 60 °C for 1 min and at 72 °C for 45 s, with a final extension at 72 °C for 5 min. The specificity and accuracy of the amplification were validated by agarose gel electrophoresis. The amplicon sizes were then verified by comparing the visible DNA bands with the DNA ladder. The sequencing for the whole exon and exon 2–5 was outsourced to the Barcode-Tagged Sequencing™ (BT-Seq™), Seoul, South Korea, and 1st Base Sequencing Plus, Apical Scientific, Malaysia respectively. Due to poor quality, 5 samples were excluded from sequencing. The resulting chromatograms were analyzed using Geneious Prime software V2022 (https://www.geneious.com/)^17^.

#### 2.2.3 Genotyping

SNP genotyping was carried out using the TaqMan assay (Thermo Fisher Scientific assay ID: C___2552981_1_) in Applied Biosystems 7500 fast real-time PCR equipment (Applied Biosystems, CA, USA). This RT-PCR method differentiates wild-type homozygous, heterozygous, and rare homozygous genotypes by analyzing the fluorescence from allele-specific, dye-labeled probes. Each assay included forward and reverse primers to amplify the target sequence and two TaqMan probes: a VIC-labeled probe for Allele 1 (C) and a FAM-labeled probe for Allele 2 (T). The PCR reaction, with a final volume of 10 µL, consisted of TaqMan genotyping master mix (2.5 µL), genotyping assay mix (0.125 µL), PCR-H_2_O (2.675 µL), and template DNA (4.7 µL). PCR conditions were 95 °C for 10 min, followed by 40 cycles of 95 °C for 15 s and 60 °C for 1 min.

#### 2.2.4 Statistical analysis

The Statistical Package for the Social Sciences (SPSS, v21.0) was utilized to analyze the demographic data gathered from the structured questionnaire and clinical parameters. The results were presented as mean ± SD for continuous variables and % (no. of part) for categorical questions. The threshold for statistical significance was set at p ≤ 0.05. After obtaining genotyping and allelic frequencies, the association analysis between the SNPs and disease outcome was conducted to determine the gene's susceptibility to the disease phenotype. For this purpose, SNPStats (https://www.snpstats.net/start.htm?) was used^18^. With the help of this tool, the association of SNPs with disease outcome and biochemical levels was analyzed which was also adjusted with age, gender, and disease response.

### 2.3 *In-silico* analyses

*In-silico* analyses were conducted to evaluate the pathogenicity of non-synonymous SNPs (nsSNPs), non-coding SNPs (UTR variants), and synonymous SNPs (sSNPs). Table 2 summarizes these analyses. Pathogenicity tools such as SIFT, PhD-SNP, Meta-SNP, PolyPhen, PANTHER, SNP&GO, and SNAP predicted the impact of nsSNPs on protein function. SIFT (sorting intolerant from tolerant) uses sequence homology and the physical characteristics of amino acids to anticipate the effects of amino acid replacement on protein function^19^. PhD-SNP is an SVM-based classifier and can predict if an SNP is naturally occurring or linked to a disease^20^. PolyPhen-2 (Polymorphism Phenotyping v2) employs PSIC software (Position-Specific Independent Counts) and several features, including sequence, phylogenetic, and structural information to characterize amino acid substitutions^21^. The PANTHER-PSEP approach uses a unique metric based on the evolutionary conservation of homologous proteins to distinguish disease-causing variants from naturally occurring ones^22^. SNP&GO uses the corresponding protein functional annotation to predict whether a variation is associated to a disease^23^ while SNAP creates sets of randomly selected SNPs and compares them to the set of query SNPs based on allele frequency^24^. Lastly, using the result of the four tools (SIFT, PANTHER, PhD SNP, and SNAP) as an eight-element feature vector as input, MetaSNP, a random forest-based binary classifier outputs the likelihood that an SNP is linked to a disease or not^23^. Stability analysis tools, including I-Mutant, INPS, and MuPro, provided ΔΔG values indicating changes in protein stability, with negative values suggesting reduced stability and positive values indicating increased stability. The NetSurf 3.0 tool assessed the exposure of amino acid sites using the relative surface accessibility (RSA) metric, considering amino acids with RSA values above 25 % as surface exposed. For non-coding SNPs, tools like polymiRTS, RegulomeDB, usDSM, and UNAfold were used to explore their regulatory impacts. This approach reflects the evolutionary balance between mutation tendencies and the need to preserve molecular structure and function. For the analysis of synonymous SNP, UNA Fold and usDSM tools were used. UNA Fold web server was applied for the folding and prediction of mRNA structure^25^. On the other hand, usDSM (undersampling scheme-based method for deleterious synonymous mutation) prediction uses a random forest classifier and 14-dimensional biology features to detect deleterious synonymous mutations^26^. Additionally, four further *in-silico* analyses were conducted to assess the potential impact of the synonymous variant rs5435. Human Splicing Finder^27^ was used to predict alterations in splice sites or splicing regulatory motifs. RegulomeDB^28^ and SNPinfo FuncPred^29^ were employed to investigate whether rs5435 lies within transcription factor binding sites, regulatory motifs, or eQTL regions. Codon usage effects were evaluated using the Kazusa Codon Usage Database^30^ alongside the tissue-specific CoCoPUTs^31^ and codon statistics resources^32^ to assess translation efficiency by comparing codon frequency (CGA vs CGG) in human sequences.

**Table 2:** Bioinformatics tools used for *in-silico* analyses.

*In silico* analyses				
For nsSNPs			For non-coding SNPs (UTR variants)	For sSNPs
Pathogenicity analysis	Stability analysis	Surface accessibility analysis		
• SIFT • Phd SNP • Meta SNP • Polyphen • Panther • SNP & GO • SNAP	• I mutant • INPS • Mu pro	• Netsurf 3.0	• polymiRTs • RegulomeDB	• usDSM • UNA Fold • HSF • RegulomeDB • SNPinfo FuncPred • CoCoPUTs

## 3 Results

### 3.1 Features of study participants

Demographic, anthropometric and biochemical features of the study participants along with significant differences between the groups are outlined in Table 3.

**Table 3:** Demographic, anthropometric and biochemical characteristics of all study participants.

Parameters	T2D (n = 127)	Control (n = 112)	P value
	Mean ± sd	Mean ± sd	
Age (years)	50.47 ± 10.69	39.79 ± 11.72	< 10^-4^
Gender	Male (60)Female (67)	Male (62)Female (50)	0.21 0.21
Height (cm)	165.96 ± 3.84	164.35 ± 6.58	0.10
Weight (Kg)	67.77 ± 5.98	65.84 ± 6.58	0.09
BMI (kg/m^2^)	24.60 ± 2.00	24.39 ± 2.25	0.58
SBP (mmHg)	125.16 ± 9.53	122.45 ± 7.85	0.09
DBP (mmHg)	83.79 ± 5.33	82.10 ± 7.37	0.15
FBS (mmol/L)	9.43 ± 3.47	5.12 ± 0.34	< 10^-5^
HbA1c (%)	9.64 ± 2.01	5.57 ± 0.30	< 10^-5^
Creatinine (mg/dL)	1.25 ± 1.22	0.67 ± 0.09	0.02
ALT (IU/L)	43.64 ± 33.05	30.67 ± 9.40	0.06

T2D: Type 2 diabetes mellitus, SBP: Systolic Blood Pressure, DBP: Diastolic Blood Pressure, BMI: Body Mass Index, FBS: Fasting blood sugar; HbA1c: Glycated hemoglobin; ALT: Alanine transaminase.

The significant difference in mean age (Table 3) between the diabetic and healthy groups indicates that age may act as a confounding variable, potentially influencing the levels of biochemical parameters. To account for this, a multivariate analysis was performed to adjust for both age and gender. The adjusted results are presented in Sup. Table S1. Although there was no significant difference in sex distribution between the two groups, sex-specific biological factors (e.g., hormonal influences) may still modulate genetic associations. Therefore, adjusting for sex helps ensure that the observed effects are not biased by such variability.

### 3.2 Genetic analysis of the *SLC2A4* gene

Genetic analysis was carried out to evaluate the impact of *SLC2A4* gene variants in T2D patients following PCR amplification and sequencing of the resulting amplicons. All 11 exons of the *SLC2A4* gene were amplified in a subset of participants (10 T2D patients and 10 healthy controls) using four primer pairs. Supplementary Fig. S1 shows the agarose gel electrophoresis images of the PCR products. Sanger sequencing was conducted, and the nucleotide sequences of the amplicons were aligned with the reference *SLC2A4* gene sequence (NG_012127.1;13,314 bp). After comparing the chromatograms, a total of 4 genetic variants were identified. The characteristics of these four variants are summarized in Table 4.

**Table 4:** Variants detected in the *SLC2A4* gene.

Nucleotide position in the reference sequence	Reference allele	Altered allele	Annotated rsID	Genomic position
5009	C	A	rs5417	5′ UTR
5039	G	A	rs5418	5′ UTR
6491	C	T	rs201878149	Intron 1
7070	T	C	rs5435	Exon 4

Comprehensive analysis of whole exon sequencing data for the *SLC2A4* gene revealed a single exonic variant, rs5435, located in exon 4. This region was amplified using primer pair 2, as detailed in Table 1. Based on the initial screening results, exon 4 was selected for further investigation in a substantial cohort, comprising 52 T2D patients and 47 healthy individuals. PCR amplification using primer pair 2, followed by Sanger sequencing, confirmed the presence of the rs5435 variant. No other genetic variants were identified in this region. Consequently, TaqMan probe-based RT-PCR was employed to determine the genotype and allele frequency of rs5435 in the remaining study participants.

### 3.3 Association studies

Allelic frequency of rs5435 analysis shown in Table 5 reveals that there was no significant difference in the distribution of the C (variant) and T (wild-type) alleles between T2D patients and healthy individuals (OR:1.37, p = 0.24). However, the odds ratio has not been adjusted for any confounding variables.

**Table 5:** Allelic frequencies of rs5435 in study participants.

Allele	Control, (n)	T2D, (n)	OR (95 % CI)	χ^2^ value	P value
T	106	103	1.00	0.13	0.13
C	118	151	1.31 (0.91–1.89)		

T2D: subpopulation with type 2 diabetes; n: count in each genotype; (%): denote percent distribution of each genotype; OR: odds ratio; CI: confidence interval; χ^2^ = chi-square.

Genotypic analysis revealed that among healthy individuals, 30.4 % were homozygous for the wild-type allele (TT), 33.9 % were heterozygous (TC), and 35.7 % were homozygous for the variant allele (CC). In comparison, among T2D patients, 20.5 % were TT, 40.2 % were TC, and 39.4 % were CC. Among the four genetic inheritance models shown in Table 6, the dominant model (TT vs TC-CC) with an odds ratio of 2.53 shows a statistically significant association (p = 0.042) with the disease. This implies that at least one copy of the C allele (heterozygous or homozygous for the variant allele) is enough to express the trait or increase the risk of the associated disease (T2D). Individuals with either one or two copies of the variant (heterozygous or homozygous) are at an elevated risk, while individuals with two normal copies (homozygous wild-type) have a lower risk of T2D.

**Table 6:** Association of genotypic frequencies of rs5435 with T2D in study participants.

Model	Genotype	Control, n (%)	T2D, n (%)	OR (95 % CI)	P value
Codominant	T/T	34 (30.4 %)	26 (20.5 %)	1.00	0.12
	T/C	38 (33.9 %)	51 (40.2 %)	2.73 (0.99–7.53)	
	C/C	40 (35.7 %)	50 (39.4 %)	2.34 (0.84–6.53)	
Dominant	T/T	34 (30.4 %)	26 (20.5 %)	1.00	0.042
	T/C–C/C	78 (69.6 %)	101 (79.5 %)	2.53 (1.01–6.36)	
Recessive	T/T-T/C	72 (64.3 %)	77 (60.6 %)	1.00	0.57
	C/C	40 (35.7 %)	50 (39.4 %)	1.26 (0.57–2.79)	
Overdominant	T/T-C/C	74 (66.1 %)	76 (59.8 %)	1.00	0.22
	T/C	38 (33.9 %)	51 (40.2 %)	1.64 (0.75–3.60)	

T2D: patients with type 2 diabetes; n: count in each genotype; (%): denote percent distribution of each genotype; OR: odds ratio; CI: confidence interval.

No significant association was observed between rs5435 and the risk of T2D in any of the other three genetic models tested: codominant (p = 0.12), recessive (p = 0.57), and overdominant (p = 0.22), even though these models displayed odds ratios greater than 1.00. In the codominant model, the T/T genotype was used as the reference due to T being the wild allele, even though this genotype had a lower frequency. Since age and gender are potential confounding variables that may influence the levels of association, the analysis presented in Table 6 was adjusted for both variables. Additionally, stratified analyses were performed separately for male (Sup. Table S2) and female (Sup. Table S3) participants to assess potential gender-specific effects. However, no significant association was found between rs5435 and T2D in either subgroup, and this lack of association remained consistent across all genetic models.

### 3.4 *In-silico* analyses of *SLC2A4* gene variants

The SNPs of the *SLC2A4* gene were collected from dbSNP and Ensembl databases. Among them 420 were non-synonymous (nsSNP), 221 were synonymous (sSNP), 1288 were intronic, 532 were 3́ UTR, and 130 were 5́ UTR variants.

#### 3.4.1 nsSNP analysis

Seven bioinformatics tools- SIFT, Phd SNP, Meta SNP, Polyphen, Panther, SNP & GO, and SNAP—were employed to identify the most deleterious nsSNPs. Based on the cutoff values of these tools, 26 missense variants were deemed deleterious (Sup. Table 4 and Supplementary Fig. S2). Out of these 26 variants, 17 were considered harmful by at least six tools. These SNPs included E225D, E409D, G141A, G170D, L352F, P165H, R169Q, R285W, R346Q, R349H, R350W, R416H, R474Q, R474W, S35F, V319E, and Y448C. The influence of these 17 missense SNPs on the protein stability was assessed using three predictive tools: I-mutant 3.0, INPS-MD, and MUpro shown in Sup. Table S5. Except for S35F and E225D, all the other SNPs were found to decrease the stability of the protein by all three tools. In addition, the surface accessibility of the SNPs was also evaluated using the NetSurf 3.0 tool, and the result is shown in Sup. Table S6.

**Fig. 2:**
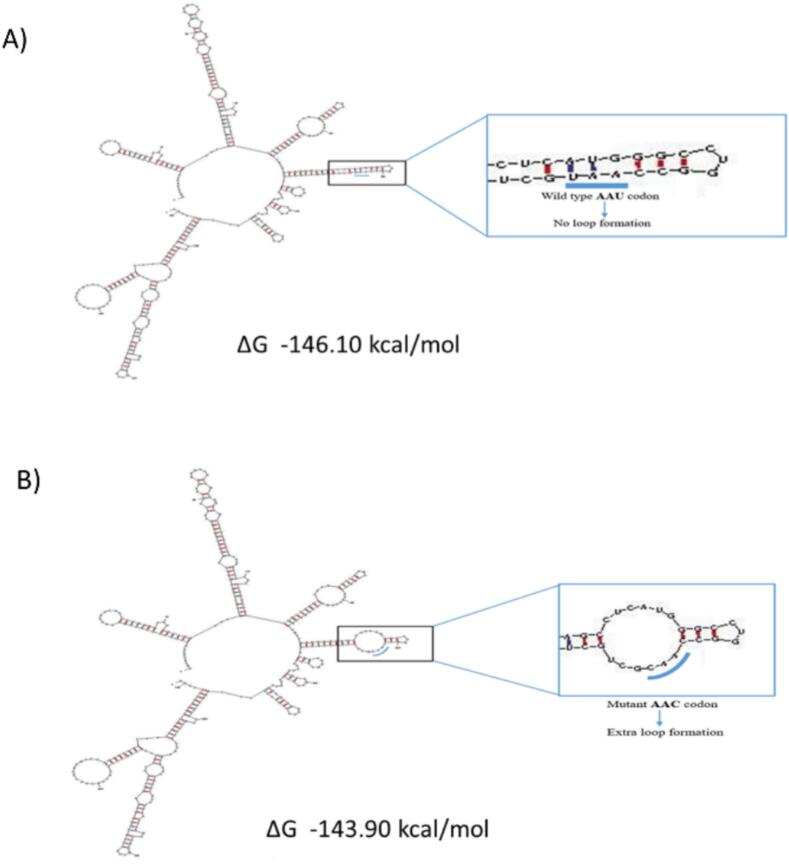
Secondary structure of the 400-nucleotide flanked GLUT4 mRNA containing rs5435, predicted by the UNAfold tool (A) Wild type allele does not create any loop, (B) rs5435 causes an extra loop in the structure.

#### 3.4.2 Untranslated region (UTR) variants analysis

Mutations in UTRs can lead to severe pathologies^33^. In analyzing the *SLC2A4* gene’s UTR region for pathogenic variants, RegulomeDB and PolymiRTS were used. Supplementary Tables S7 and 8 show the results obtained from regulomeDB and polymiRTS tools respectively. No variant was common between both tools. In this study, after sequencing *SLC2A4*, two 5′ UTR variants, rs5417 and rs5418, were found in the initial study participants. RegulomeDB identified them with low scores, while PolymiRTS showed no results for them, implying they might not be pathogenic.

#### 3.4.3 *S*ynonymous variant rs5435 analysis

Secondary structure prediction by the UNA Fold tool reveals that this variant can create an extra loop in the mRNA structure shown in Fig. 2. The rs5435 variant introduces an additional loop in the mRNA secondary structure. Compared to the wild type of structure, which has a ΔG value of −146.10 kcal/mol and lacks this loop, the variant's structure has a ΔG value of −143.90 kcal/mol. As a result, the rs5435 variant leads to a less stable secondary structure. In a recent study, the usDSM tool has been introduced for identifying harmful synonymous mutations^26^. According to this tool, the rs5435 variant is categorized as deleterious.

##### 3.4.3.1 Functional analyses of rs5435

Beyond secondary structure and usDSM-based deleteriousness prediction, additional *in silico* analyses provided deeper insights. Human Splicing Finder analysis found no significant disruption of known splice donor/acceptor sites or regulatory splicing motifs due to rs5435. RegulomeDB returned a score of 7, indicating minimal evidence for regulatory function at this locus. SNPinfo FuncPred analysis similarly showed no predicted transcription factor binding changes or miRNA-related effects. Importantly, codon usage analysis using CoCoPUTs and the Codon Statistics Database revealed that the CGA codon (used by the rs5435 variant) is significantly rarer (RSCU ≈ 6.2) compared to the most common arginine codon CGG (RSCU ≈ 11.7). This disparity in usage suggests that rs5435 may reduce translational speed, potentially affecting co-translational protein folding or expression levels. A summary of the predicted functional impacts of rs5435 from all *in silico* tools is presented in Table 7**.**

**Table 7:** Summary of *in silico* functional predictions for the rs5435 variant using multiple tools.

Analysis	Tool	Result	Interpretation
Splice motif disruption	Human Splicing Finder	No gain/loss of ESE/ESS motifs; no splice site impact	Unlikely to affect pre-mRNA splicing
Regulatory element disruption	RegulomeDB (score = 7)	No ChIP-seq, DNase, or eQTL evidence	No support for TF-binding site overlap or eQTL
Transcription factor binding	SNPinfo FuncPred	No change in TFBS, miRNA-binding, or RBP motifs	No predicted transcriptional regulation impact
Codon usage & translation efficiency	Human codon usage tables,CoCoPUTs, Codon Statistics Database	CGA (rare) = 6.2 %; CGG (normal) = 11.4–11.7 %; CGA ∼ half as frequent	Rare codon → potential ribosome pausing, slower translation, altered folding

ESE: exonic splicing enhancer, ESS: exonic splicing silencer, eQTL: expression Quantitative Trait Locus, TFBS: Transcription Factor Binding Site, RBP: RNA-binding protein.

## 4 Discussion

This study presents the first investigation of SLC2A4 (GLUT4) gene variants and their association with type 2 diabetes (T2D) in the Bangladeshi population, identifying rs5435 as a potential risk variant. While previous studies in South Indian and Chinese populations have reported the T allele of rs5435 to be associated with increased T2D risk^9^, ^11^, our findings indicate that the C allele may confer risk in the Bangladeshi cohort under a dominant genetic model. This highlights possible population-specific effects of rs5435, underscoring the genetic diversity within South Asian populations. Additionally, *in silico* analyses suggest that this synonymous variant may alter mRNA structure and translational efficiency, providing functional insights into its potential role in T2D pathogenesis.

Being one of the most prevalent non-communicable diseases globally, diabetes is now considered a major health challenge of the 21st century. While in 2000 it was estimated that around 151 million people were living with diabetes worldwide, in 2025 this number has risen to a staggering height of 590 million^1^. Due to the immense adverse impact of T2D on the quality of human life and the economic burden on societies and governments worldwide, there has always been a growing concern and effort for the betterment of its diagnosis and treatment. In this context, the genetics of T2D is crucial to understand as numerous genetics studies have already established a very strong role of genetics in the development of the disease. GLUT4 variants that are associated with T2D^2^, ^5^, ^9^ have been identified across different ethnic groups. However, to date, there is no report on the association of GLUT4 genetics with the risk of T2D in the Bangladeshi population.

This study aimed to explore the relationship between GLUT4 encoding *SLC2A4* gene variants, particularly rs5435, and the risk of T2D within the Bangladeshi population. Employing a combination of *in silico* and *in vitro* methods, genetic variants of the *SLC2A4* gene were investigated in 239 individuals. The primary focus of this work was to explore the whole exon of GLUT4 transporter to identify any recorded or novel genetic variations present in our population. This way, the entire genetic landscape of the GLUT4 transporter was analyzed instead of a particular SNP. The initial sequencing data revealed the presence of 4 SNPs including rs5435 which is an exonic variant. Previous studies in diverse populations have reported this GLUT4 gene variant in the context of type 2 diabetes susceptibility^2^, ^3^, ^4^, ^5^, ^6^, ^8^, ^9^. However, to our knowledge, only one study, conducted in a South Indian population, has reported a statistically significant association between the rs5435 variant and T2D^9^. A second South Indian study also examined GLUT4 but did not find an association with rs5435^8^. Based on our sequencing result and its prior implication in one T2D study^9^, rs5435 was selected for detailed genotyping and association analysis in our study population. A TaqMan probe-based RT-PCR assay was conducted to determine the allelic and genotyping frequencies of rs5435 in the remaining study participants. Statistical analysis showed no significant overall association between the C allele and T2D risk (OR: 1.37, p = 0.24). However, under a dominant genetic model (TT vs. TC/CC), a significant association was observed (OR: 2.53, p = 0.042), suggesting that carrying at least one copy of the variant C allele may increase susceptibility to T2D. This could mean that the variant is strong enough to have effect on the disease in heterozygous individuals (having one copy of the SNP). Although the effect size may appear modest, this finding warrants further investigation in larger studies to better understand the true impact of this SNP on T2D risk in the Bangladeshi population. Our findings are also unique in the sense that previously this polymorphism was found to be associated with diabetes in a South Indian population where they showed that the T allele of rs5435 was associated with a high risk of T2D^9^. Again, in another study when the association of rs5435 with blood glucose and insulin was analyzed in a Chinese population, it was found that fasting blood glucose and fasting insulin were higher in women with the TT genotype^11^. On the contrary, this study has shown that the presence of at least one copy of the variant C allele may increase susceptibility to T2D in the Bangladeshi population.

Additionally, although no significant sex difference was observed in the overall distribution, gender-wise genetic association analyses were conducted to explore potential sex-specific effects. This approach helps uncover associations that may manifest differently in males and females due to biological factors like hormonal profiles and metabolic responses and ensures the identification of associations that might not be evident in the combined cohort. The marginal significance observed in the dominant model likely reflects a cumulative effect across the sexes. In contrast, the lack of association in gender-wise analyses is attributed to reduced statistical power as the sample size of each subgroup was much smaller than the overall cohort. Smaller subgroup analyses are more susceptible to Type II error, where true associations may go undetected, and are generally considered less reliable without validation in larger cohorts^34^.

The SNP rs5435 is a synonymous variant. Earlier studies have argued that sSNPs are as likely to be pathogenic as non-synonymous variants. They have been implicated in many diseases, including pulmonary sarcoidosis, attention deficit or hyperactivity disorder, and cancer. These variants can disrupt transcription, splicing, co-translational folding, and mRNA stability, and cause a plethora of other functionally relevant changes^35^. Therefore, in this study, the functional and structural impact of rs5435 on GLUT4 was predicted using bioinformatics tools. *In-silico* analyses revealed that the rs5435 variant might create an extra loop in the mRNA's secondary structure, potentially reducing translational efficiency and protein expression^36^, ^37^. This altered structure was thermodynamically less stable, indicated by a higher free energy compared to the wild type. Previous research has shown that such structural changes result in a reduction of translational rate, co-translational protein folding, and reduced protein expression, and finally may cause the disease associated with that protein^35^. Our results also suggested that rs5435 may exert similar disruptions of the function of GLUT4 by creating structural changes. Any variation that reduces the availability or activity of GLUT4, such as a decrease in its protein levels due to altered mRNA stability, could result in impaired glucose uptake, contributing to insulin resistance and hyperglycemia, both of which are hallmarks of T2D. To further evaluate the potential functional role of rs5435, the *in silico* analysis was extended beyond mRNA structure. Splicing analysis using HSF found no evidence that rs5435 alters exonic splicing regulatory elements. RegulomeDB and SNPinfo tools also did not support a regulatory or transcription factor binding role. However, codon usage (CGA vs CGG) analysis revealed that CGA is a low-frequency arginine codon in humans, which may slow translation elongation at this site. Such delays can disrupt co-translational folding, potentially reducing GLUT4 functionality. These findings align with literature suggesting that rare codon usage at key residues may alter protein yield and structure^37^, ^38^. This mechanism provides a plausible functional link between a silent SNP and altered protein phenotype, independent of regulatory element disruption. In addition, several biological pathways including AMPK signaling, FoxO signaling, insulin signaling, adipocytokine signaling, diabetic cardiomyopathy, and insulin resistance where GLUT4 plays a role may get affected by altered GLUT4 expression and function. GLUT4 plays a crucial role in maintaining glucose homeostasis by facilitating glucose uptake into cells in response to insulin signaling. The sSNP rs5435 may affect transcriptional regulation of SLC2A4, leading to reduced GLUT4 levels in muscle and adipose tissue. This variant may also affect GLUT4′s interaction with insulin signaling, reducing its membrane translocation efficiency. As a result, individuals carrying the risk allele might exhibit reduced GLUT4-mediated glucose uptake, thereby increasing susceptibility to T2D.

In addition, the missense and UTR variants of the *SLC2A4* gene were thoroughly analyzed in this work by using various databases and predictive tools. Among the reported non-synonymous SNPs (nsSNPs), 26 were identified as deleterious. These nsSNPs can influence protein stability by altering the native amino acid, affecting protein folding and function. Tools such as SIFT, Polyphen, and SNAP were employed to predict the deleterious effects of these nsSNPs. Several different tools were combined for the initial screening to ensure a robust and accurate prediction with increased reliability. Moreover, NetSurf 3.0 was applied to identify exposed and buried residues of the protein, highlighting the functional importance of amino acid positions in ligand binding, structural stability, and protein–protein interactions. Based on these findings a guideline can be prepared to detail all the deleterious SNPs within GLUT-4 which can potentially increase the risk of diabetes. Taken together, these predicted deleterious variants may compromise multiple aspects of GLUT4 function such as protein folding, translocation, and membrane localization, ultimately impairing insulin–stimulated glucose uptake and contributing to T2D pathogenesis through combined effects. Mechanistic studies frequently highlight that defective insulin–stimulated GLUT4 translocation is a hallmark of insulin resistance^38^, ^39^**,** while post–translational modifications are known to regulate GLUT4 sorting and membrane delivery^40^, ^41^**.** Moreover, in-vivo evidence shows that reduced GLUT4 expression significantly impairs glycemic control^42^, ^43^**.**

In summary, this study, combining both *in silico* and *in vitro* analyses, is the first in Bangladesh to sequence the *SLC2A4* gene in our population and explore the association of the identified rs5435 variant with T2D risk. Furthermore, with the help of bioinformatics analyses, the results shed light on how rs5435 might interfere with the function of the GLUT4 transporter.

The study has several limitations. While the study focused on the rs5435 variant of the SLC2A4 gene, it did not investigate other genetic variants involved in insulin signaling or glucose metabolism, which may also contribute to type 2 diabetes (T2D) risk through additive or interactive effects. Additionally, lifestyle and environmental factors—such as dietary habits, physical activity, socioeconomic status, and family history—were not systematically collected or adjusted for in this study. These variables are known to independently affect T2D risk and may confound observed genetic associations^44^, ^45^, ^46^. As a result, the inability to control these non-genetic factors restricts our potential to adequately untangle genetic contributions and environmental influences. Future studies incorporating these dimensions are needed to better model the interplay between genetic susceptibility and modifiable risk factors. Besides, while the sample size in this study is adequate for preliminary analysis, we acknowledge that the relatively small sample size could limit the statistical power of the study and its ability to detect subtle associations. The p-value for the allelic frequency of rs5435 (p = 0.24) and the confidence intervals for the odds ratios indicate that the effect sizes might not be large enough to reach strong statistical significance with the current sample size. Therefore, these findings should be interpreted with caution, and further studies with larger and more diverse cohorts are needed to validate these associations and assess their broader applicability. Additionally, while recruiting patients from a single tertiary hospital may introduce some selection bias, this center admits patients from across Bangladesh, offering a diverse and well-characterized cohort. The high-quality clinical data enhances the reliability of phenotype-genotype associations, which is critical for studying complex diseases like T2D. We acknowledge the limitation of our cross-sectional design and plan future longitudinal studies to better assess how genetic variants influence T2D progression over time. Lastly, assessing whether particular SNP combinations are enriched in T2D patients or impact gene function collectively could provide more insight into the gene’s role in disease pathogenesis, which represents an important direction for future research.

## 5 Conclusion

This study identified a significant association between the rs5435 variant of the *SLC2A4* gene and T2D risk in the Bangladeshi population, particularly in the dominant genetic model. Although rs5435 is a synonymous variant, its potential impact on mRNA structure and translation underscores the importance of considering such variants in genetic studies. This is particularly novel because the functional relevance of synonymous variants is often overlooked. This study draws attention to the need to explore the potential roles of these variants in disease development. The study's focus on the Bangladeshi population is significant because genetic studies on T2D often prioritize Western or more commonly studied populations. By concentrating on a South Asian population, the research offers valuable insights into the genetic predispositions of T2D in an underrepresented group. Moreover, if confirmed, the association of the rs5435 variant with T2D risk could lead to a better understanding of the genetic architecture of the disease, especially in South Asian populations. This might eventually help in developing more tailored genetic testing and risk assessment strategies for T2D in the Bangladeshi population. However, further investigations are required to validate these findings in larger cohorts with enhanced statistical power and robustness. Overall, the findings could pave the way for more inclusive genetic studies and offer insights into previously underexplored genetic factors in complex diseases like T2D.

## Funding

This study was supported by the Dhaka University Centenary Grant (2nd level) 2020–2021 (Reg/Adm-3/70928) awarded to Dr. Tahirah Yasmin.

## CRediT authorship contribution statement

**Mohammad Mamunur Rashid:** Writing – original draft, Investigation, Formal analysis. **Mohammad Sayem:** Supervision, Investigation. **Maisha Adiba:** Investigation, Formal analysis. **Abdullah Al Saba:** Supervision, Methodology. **A.H.M. Nurun Nabi:** Writing – review & editing, Supervision, Conceptualization. **Tahirah Yasmin:** Writing – review & editing, Supervision, Funding acquisition, Conceptualization.

## Declaration of competing interest

The authors declare that they have no known competing financial interests or personal relationships that could have appeared to influence the work reported in this paper.
