# Supplementary material for: Whole exon screening of SLC2A4 gene and the association of rs5435 with type 2 diabetes in a Bangladeshi case-control study
Source: J Genet Eng Biotechnol. 2025 Jul 10;23(3):100534. doi: 10.1016/j.jgeb.2025.100534 (PMC12275759; doi:10.1016/j.jgeb.2025.100534)
Supplement: Supplementary Data 1 [file mmc1.docx]

**Supplementary files**

**Supplementary Table 1:** Adjusted dataset of the demographic, anthropometric and biochemical parameters of study participants.

| **Parameters** | **T2D (n=62)** | **Control (n=57)** | **P value** |
| --- | --- | --- | --- |
| Age (years) | 50.5 ± 10.7 | 37.8 ± 11.1 | 2 × 10^-7^ |
| Height (cm) | 165.9 ± 3.8 | 165.6 ± 7.0 | 0.79 |
| Weight (Kg) | 67.8 ± 5.98 | 63.6 ± 6.21 | 10^-3^ |
| BMI (kg/m2) | 24.6 ± 2.0 | 23.1 ± 1.37 | 10^-4^ |
| SBP (mmHg) | 125.1 ± 9.5 | 121.6 ± 8.6 | 0.05 |
| DBP (mmHg) | 83.8 ± 5.33 | 81.1 ± 8.26 | 0.04 |
| FBS (mmol/L) | 9.43 ± 3.48 | 5.11 ± 0.35 | 3.36 × 10^-11^ |
| HbA1c (%) | 9.64 ± 2.01 | 5.55 ± 0.29 | 2.0 × 10^-16^ |
| Creatinine (mg/dL) | 1.25 ± 1.22 | 0.66 ± 0.098 | 0.06 |
| ALT (IU/L) | 43.6 ± 33.1 | 29.7 ± 8.13 | 0.12 |

**Table 2:** Association of genotypic frequencies of rs5435 with T2D in male study participants

| **Model** | **Genotype** | **Control, n (%)** | **T2D, n (%)** | **OR (95% CI)** | **P value** |
| --- | --- | --- | --- | --- | --- |
| Codominant | T/T | 25 (40.3%) | 17 (28.3%) | 1.00 | 0.22 |
|  | T/C | 20 (32.3%) | 22 (36.7%) | 2.11 (0.70-6.39) |  |
|  | C/C | 17 (27.4%) | 21 (35%) | 2.54 (0.80-8.02) |  |
| Dominant | T/T | 25 (40.3%) | 17 (28.3%) | 1.00 | 0.089 |
|  | T/C-C/C | 37 (59.7%) | 43 (71.7%) | 2.30 (0.86-6.13) |  |
| Recessive | T/T-T/C | 45 (72.6%) | 39 (65%) | 1.00 | 0.27 |
|  | C/C | 17 (27.4%) | 21 (35%) | 1.73 (0.65-4.64) |  |
| Overdominant | T/T-C/C | 42 (67.7%) | 38 (63.3%) | 1.00 | 0.53 |
|  | T/C | 20 (32.3%) | 22 (36.7%) | 1.35 (0.53-3.48) |  |

**Table 3:** Association of genotypic frequencies of rs5435 with T2D in female study participants

| **Model** | **Genotype** | **Control, n (%)** | **T2D, n (%)** | **OR (95% CI)** | **P value** |
| --- | --- | --- | --- | --- | --- |
| Codominant | T/T | 9 (18%) | 9 (13.4%) | 1.00 | 0.29 |
|  | T/C | 18 (36%) | 29 (43.3%) | 9.98 (0.22-443.8) |  |
|  | C/C | 23 (46%) | 29 (43.3%) | 3.15 (0.07-138.8) |  |
| Dominant | T/T | 9 (18%) | 9 (13.4%) | 1.00 | 0.27 |
|  | T/C-C/C | 41 (82%) | 58 (86.6%) | 5.53 (0.16-187.6) |  |
| Recessive | T/T-T/C | 27 (54%) | 38 (56.7%) | 1.00 | 0.5 |
|  | C/C | 23 (46%) | 29 (43.3%) | 0.52 (0.07-3.69) |  |
| Overdominant | T/T-C/C | 32 (64%) | 38 (56.7%) | 1.00 | 0.15 |
|  | T/C | 18 (36%) | 29 (43.3%) | 4.03 (0.56-29.20) |  |

**Supplementary Table 4:** List of 26 missense variants identified as deleterious by different bioinformatics tools.

| **rs ID** | **change** | **rs ID** | **change** |
| --- | --- | --- | --- |
| rs146828718 | A387T | rs200651340 | R474W |
| rs150621510 | E225D | rs376636615 | S35F |
| rs202200041 | E237K | rs201532628 | V160A |
| rs140608377 | E409D | rs377371372 | V160M |
| rs371607018 | F222I | rs147155858 | V319E |
| rs369328286 | G141A | rs375714683 | V407M |
| rs140466829 | G170D | rs201686153 | Y448C |
| rs367617703 | L352F | rs202163530 | I289T |
| rs369638698 | P165H | rs35198331 | S55R |
| rs138015791 | P287S | rs70937028 | R474Q |
| rs138717844 | R346Q | rs146787418 | R169Q |
| rs142724012 | R349H | rs140743598 | R285W |
| rs144431498 | R350W | rs144216820 | R416H |

**Supplementary Table 5**: Effect of the 17 nsSNPs on the protein’s stability predicted by different tools.

| **Variant** | **I mutant ΔΔG** | **Stability** | **INPS ΔΔG** | **Stability** | **Mu pro ΔΔG** | **Stability** |
| --- | --- | --- | --- | --- | --- | --- |
| E225D | -0.29 | Decrease | -0.80 | Decrease | 0.12 | Increase |
| E409D | -0.28 | Decrease | -0.85 | Decrease | -1.41 | Decrease |
| G141A | -0.73 | Decrease | -0.10 | Decrease | -1.16 | Decrease |
| G170D | -0.85 | Decrease | -0.81 | Decrease | -0.69 | Decrease |
| L352F | -1.01 | Decrease | -0.80 | Decrease | -1.57 | Decrease |
| P165H | -1.48 | Decrease | -0.86 | Decrease | -1.07 | Decrease |
| R169Q | -0.55 | Decrease | -1.31 | Decrease | -0.55 | Decrease |
| R285W | -0.18 | Decrease | -0.23 | Decrease | -0.34 | Decrease |
| R346Q | -0.61 | Decrease | -1.28 | Decrease | -0.66 | Decrease |
| R349H | -1.08 | Decrease | -1.10 | Decrease | -1.04 | Decrease |
| R350W | -0.19 | Decrease | -0.69 | Decrease | -0.64 | Decrease |
| R416H | -1.1 | Decrease | -0.82 | Decrease | -0.40 | Decrease |
| R474Q | -1.02 | Decrease | -1.13 | Decrease | -1.13 | Decrease |
| R474W | -0.37 | Decrease | -0.26 | Decrease | -1.03 | Decrease |
| S35F | -0.05 | Decrease | 0.60 | Increase | -0.11 | Decrease |
| V319E | -1.06 | Decrease | -1.24 | Decrease | -0.73 | Decrease |
| Y448C | -1.08 | Decrease | -1.59 | Decrease | -0.18 | Decrease |

Unit of ΔΔG is kcal/mole

**Supplementary Table 6:** Predicted surface accessibility of amino acid changes using NetSurf 3.0

| **Change** | **RSA** | **Status** | **Change** | **RSA** | **Status** |
| --- | --- | --- | --- | --- | --- |
| E225D | 0.10 | B | R350W | 0.22 | B |
| E409D | 0.14 | B | R416H | 0.05 | B |
| G141A | 0.01 | B | R474Q | 0.38 | B |
| G170D | 0.16 | B | R474W | 0.38 | B |
| L352F | 0.04 | B | S35F | 0.10 | B |
| P165H | 0.11 | B | V319E | 0.14 | B |
| R169Q | 0.09 | B | Y448C | 0.45 | E |
| R285W | 0.19 | B | R349H | 0.10 | B |
| R346Q | 0.73 | E |  |  |  |

RSA = relative surface accessibility, B = buried, E = exposed.

Amino acids with RSA values exceeding 25% were deemed to be on the surface.

**Supplementary Table 7:** UTR variants analysis by regulomeDB.

| **Variant ID** | **Chromosomal position** | **Alleles** | **MAF** | **Variant Type** | **probability** |
| --- | --- | --- | --- | --- | --- |
| rs5417 | 17:7281743 | C/A/T | 0.424(A) | 5' UTR | 0.70497 |
| rs5417 | 17:7281743 | C/A/T | 0.424(A) | 5' UTR | 0.70497 |
| rs5418 | 17:7281773 | G/A | 0.424(A) | 5' UTR | 0.60906 |
| rs560238897 | 17:7287118 | T/C/G | 0.345(C) | 3' UTR | 0.17627 |
| rs560238897 | 17:7287118 | T/C/G | 0.345(C) | 3' UTR | 0.17627 |
| rs532256398 | 17:7287138 | T/G | 0.023 (G) | 3' UTR | 0.45917 |

MAF = minor allele frequency

**Supplementary Table 8:** UTR variants analysis by polymiRTS.

| **Variant ID** | **Chr. Bp** | **Alleles** | **Global MAF** | **SNP** | **Functional Class** | **Probability** |
| --- | --- | --- | --- | --- | --- | --- |
| rs138258204 | 17:7288026 | G/A | 0.003 (A) | 3'UTR | C | 0.60 |
| rs150460962 | 17:7287174 | G/A | 0.001 (A) | 3'UTR | C | 0.34 |
| rs181568851 | 17:7287185 | C/T | < 0.001 (T) | 3'UTR | C | 0.17 |
| rs185947659 | 17:7287315 | G/A | 0.002 (A) | 3'UTR | C | 0.18 |
| rs189275417 | 17:7287745 | T/C | < 0.001 (C) | 3'UTR | C | 0.13 |
| rs5425 | 17:7287270 | A/C | < 0.001 (C) | 3'UTR | C | 0.34 |
| rs72556549 | 17:7286807 | C/T | < 0.001 (T) | 3'UTR | C | 0.13 |
| rs78907614 | 17:7286935 | A/C | < 0.001 (C) | 3'UTR | C | 0.13 |
| rs80094330 | 17:7287543 | C/T | 0.005 (T) | 3'UTR | C | 0.079 |
| rs9894700 | 17:7287742 | C/A/T | 0.011 (T) | 3'UTR | C | 0.134 |

MAF = minor allele frequency, functional class C means derived allele creates a new miRNA binding site

**Supplementary Figures**


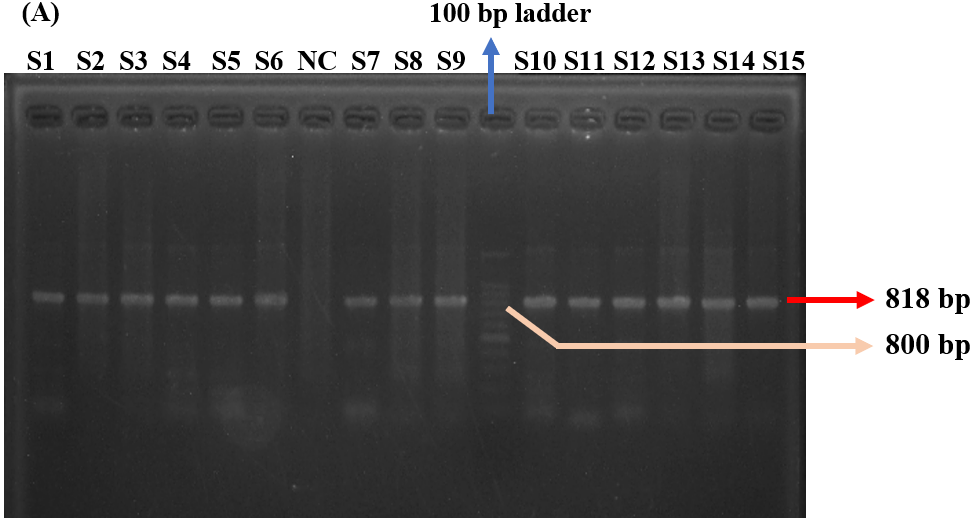


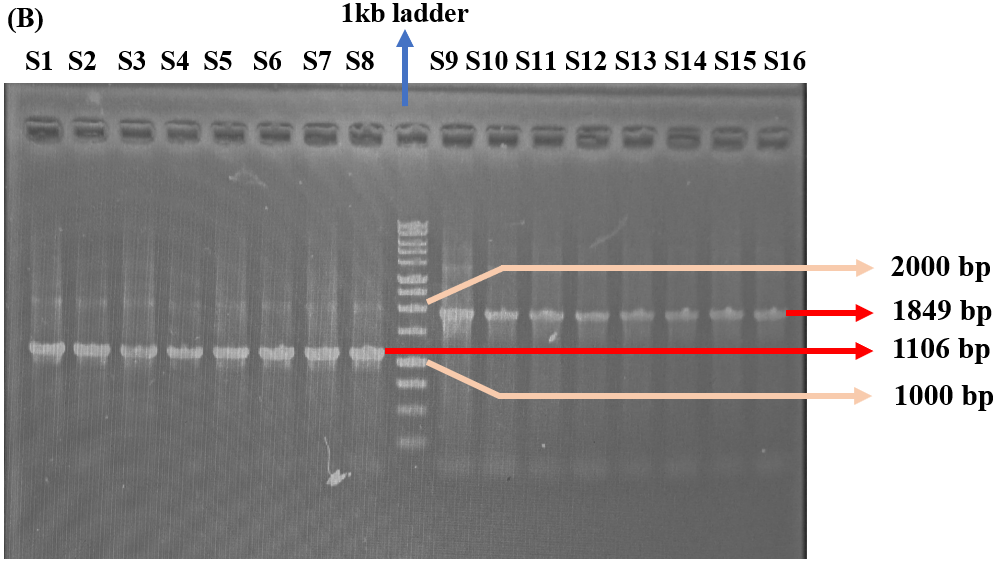


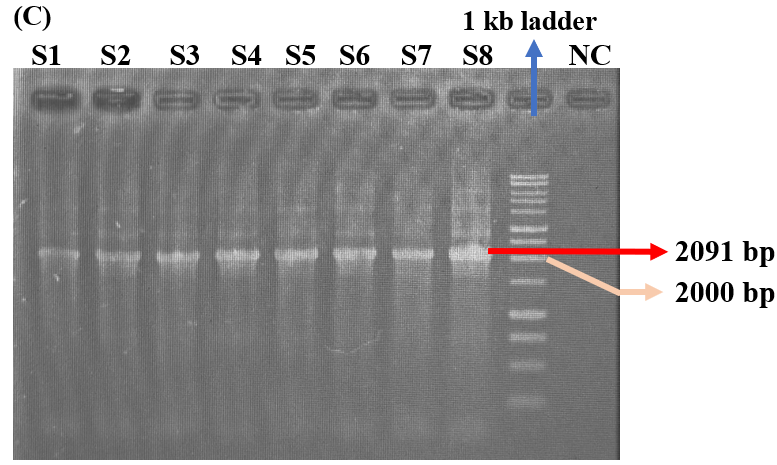


**Supplementary Figure 1:** Agarose gel electrophoresis of PCR products using *SLC2A4* gene specific primer sets. (A) Exon1 amplicon (818bp) from primer pair 1. (B) Exon2-exon5 amplicon (1106bp, left side of the gel) and exon 6-exon 10 amplicon (1849bp, right side of the gel) from primer pair 2 and 3. (C) Exon11 amplicon (2091bp) from primer pair 4. A 1kb DNA ladder determined band sizes. Samples are labeled S1-S16, with NC as the negative control.


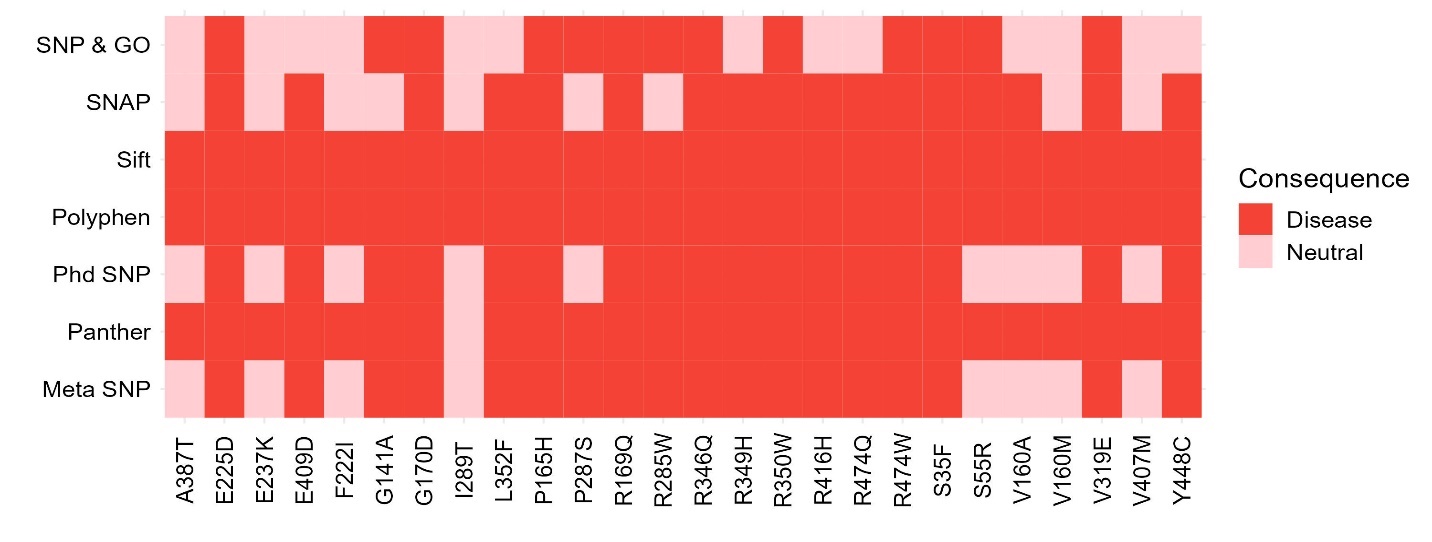


**Supplementary Figure 2:** Heatmap demonstrating the prediction of deleteriousness of nsSNPs by seven different tools. The dark tiles represent “Deleterious”, and the light tiles represent “Neutral” or “Benign” prediction for each nsSNP.
